# *SLC6A3* Polymorphism Predisposes to Dopamine Overdose in Parkinson's Disease

**DOI:** 10.3389/fneur.2018.00693

**Published:** 2018-08-21

**Authors:** Brian D. Robertson, Abdullah S. Al Jaja, Alex A. MacDonald, Nole M. Hiebert, Ruzbeh Tamjeedi, Ken N. Seergobin, Ute I. Schwarz, Richard B. Kim, Penny A. MacDonald

**Affiliations:** ^1^Schulich School of Medicine & Dentistry, University of Western Ontario, London, ON, Canada; ^2^Brain and Mind Institute, University of Western Ontario, London, ON, Canada; ^3^Department of Neuroscience, University of Western Ontario, London, ON, Canada; ^4^Department of Medicine, Undergraduate Faculty of Medicine, University of Toronto, Toronto, ON, Canada; ^5^Department of Physiology and Pharmacology, University of Western Ontario, London, ON, Canada; ^6^Faculty of Law, University of Ottawa, Ottawa, ON, Canada; ^7^Division of Clinical Pharmacology, Department of Medicine, Schulich School of Medicine & Dentistry, University of Western Ontario, London, ON, Canada; ^8^Department of Clinical Neurological Sciences, Schulich School of Medicine & Dentistry, University of Western Ontario, London, ON, Canada

**Keywords:** Parkinson's disease, polymorphism, *SLC6A3*, overdose, dopamine, encoding

## Abstract

In Parkinson's disease (PD), cognitive functions mediated by brain regions innervated by ventral tegmental area (VTA) worsen with dopamine replacement therapy, whereas processes relying on regions innervated by the substantia nigra pars compacta (SNc) improve. The *SLC6A3* gene encodes the dopamine transporter (DAT). The common 9R polymorphism produces higher DAT concentrations and consequently lower baseline dopamine than *SLC6A3* wildtype. Whether *SLC6A3* genotype modulates the effect of dopaminergic therapy on cognition in PD is not known. We investigated the effect of dopaminergic therapy and *SLC6A3* genotype on encoding and recall of abstract images using the Aggie Figures Learning Test in PD patients. Encoding depends upon brain regions innervated by the VTA, whereas recall is mediated by widespread brain regions, a number innervated by the SNc. We found that dopaminergic therapy worsened encoding of abstract images in 9R carriers only. In contrast, dopaminergic therapy improved recall of abstract images in all PD patients, irrespective of *SLC6A3* genotype. Our findings suggest that 9R-carrier PD patients are more predisposed to dopamine overdose and medication-induced impairment of cognitive functions mediated by VTA-innervated brain regions. Interestingly, PD patients without the 9R polymorphism did not show such an impairment. *SLC6A3* genotype does not modulate the dopaminergic therapy-induced improvement of functions mediated by SNc-innervated regions in PD patients.

## Introduction

Parkinson's disease (PD) is a neurodegenerative disorder characterized by substantial dopamine-producing neuron loss in the substantia nigra pars compacta (SNc) with relative sparing of dopamine-producing neurons in the ventral tegmental area (VTA). The SNc principally supplies dopamine to the dorsal striatum (DS), comprising the bulk of the putamina and caudate nuclei. The ensuing depletion of dopamine to the DS produces the cardinal PD motor manifestations of rigidity, tremor, and bradykinesia (1). Dopamine replacement medication reliably and effectively improves these DS-mediated motor symptoms in PD (2).

Although motor symptoms are uniformly improved by dopaminergic therapy in PD, distinct cognitive functions are dissimilarly affected by dopamine replacement therapy. Some cognitive functions are worsened whereas others are ameliorated or redressed by dopaminergic medication (2). Functions that depend upon brain regions receiving dopamine from the relatively-spared VTA have been found quite consistently to be worsened by exogenous dopamine therapy (2). The detrimental effects of dopaminergic therapy on cognition have been attributed to an overdose of dopamine in VTA-innervated brain regions that receive normal or near-normal dopamine in PD (2–5). These VTA-innervated regions include the ventral striatum (VS)—comprising the nucleus accumbens and most ventral parts of putamen and caudate nuclei, orbitofrontal cortex, prefrontal and limbic cortical regions, including the hippocampus (2–5). In contrast, there is now an ample literature suggesting that cognitive functions that depend upon DS, or cortical regions reciprocally connected to DS, are improved by dopaminergic therapy (2–5).

Of importance to the current study, learning, in its various forms, is mediated by VTA-innervated regions such as the VS, hippocampus, and medial frontal cortex (6–9). This function is normal at baseline and worsened by dopaminergic medications in PD (2, 10–13) and in healthy adults (14, 15). On the other hand, decision making and memory retrieval implicate DS. These functions are impaired at baseline and improve with dopaminergic therapy in PD (2, 4, 16–18).

Dopamine transporter (DAT), encoded by gene *SLC6A3*, is a membrane transporter protein that resorbs synaptic dopamine. *SLC6A3* is abundant in the striatum, midbrain, and hippocampus, but scarce in the prefrontal cortex (19)—where synaptic dopamine is degraded primarily by catechol-*O*-methyltransferase (COMT). In the *SLC6A3* gene, a 40-base pair variable nucleotide tandem repeat element exists, with 9-(9R) and 10-repeat (10R) forms being most prevalent (20). Recent meta-analyses (21, 22) that analyzed data from studies in which positron emission tomography and single photon emission computed tomography was used have clarified that presence of the *SLC6A3* 9R allele causes higher DAT levels than 10R-homozygosity. Hence, 9R-carriers are expected to have lower baseline dopamine concentrations compared to 10R-homozygotes.

In 9R carriers, increased expression of DAT, and consequently lower concentrations of dopamine at baseline, are expected to enhance the ratio between phasic, pulsatile, dopamine bursts related to events such as reward, positive feedback, or behavior, and tonic, basal dopamine release that occurs at rest. This enhanced signal-to-noise ratio is expected to result in more efficient signaling and potentially improved learning and performance. However, given that this superior performance is expected on the basis of lower tonic, basal dopamine, 9R carriers are predicted to be more susceptible to overdose effects of exogenous dopamine.

In line with the hypothesis that 9R carriers have more efficient dopamine signaling and potentially improved performance, 9R carriers have been shown to have enhanced activity in bilateral striatum upon the reception of positive feedback (23, 24). Dreher et al. also found that 9R carriers had greater reactivity in the midbrain and lateral PFC upon the reception of reward and, further, showed enhanced reactivity in DS and VS during reward anticipation (25). Further, in a PET study of habitual smokers, increased smoking–related VS reactivity—hypothesized to be due to larger phasic dopamine bursts in 9R carriers, who have lower tonic synaptic dopamine concentrations—was seen in 9R carriers relative to 10R/10R homozygotes (26). 9R carriers, as compared to 10R/10R homozygotes, have also been shown to evidence a larger frontoparietal, novelty-dependent electroencephalographic response during the presentation of auditory cues signaling a task switch during a test of cognitive flexibility (27). These results suggest that 9R carriers are more sensitive to phasic increases in dopamine in the striatum.

The effect of *SLC6A3* gene polymorphisms on cognition in PD patients has scarcely been investigated (28). In the lone investigation of this *SLC6A3* polymorphism in PD using neuroimaging, patients carrying a 9R allele exhibited less activation than their 10R homozygous counterparts in caudate nucleus and prefrontal and premotor cortices when planning and executing a set-shift (28). This is in contradistinction to studies with healthy controls in which 9R-carriers consistently exhibit greater cognition-related neural activation relative to 10R-homozygotes using neuroimaging (24, 25, 29, 30).

To our knowledge, how *SLC6A3* polymorphisms affect response to dopaminergic therapy has not been examined in PD. In healthy controls, dopaminergic therapy *reduced* abilities of healthy 9R-carriers relative to 10R-homozygotes to maximize earnings by learning and adapting to the playing style of their opponents in a competitive task (31). In contrast, dopaminergic therapy *improved* cognitive flexibility, a DS-mediated function (2, 32), in healthy 10R-homozygotes but not 9R-carriers (27). These findings present the intriguing possibility that *SLC6A3* genotype interacts with the now well-described differential medication effects in VTA-innervated brain regions vs. DS (2, 3).

### Current study

This study was designed to investigate the role of the *SLC6A3* polymorphism on memory encoding and retrieval in PD patients on and off dopaminergic medication. Identifying gene-medication interactions for cognitive symptoms in PD patients would be important from both a clinical and basic science standpoint. Clinically, identifying genes that interact with medication to differentially affect cognition could lead to more customized treatment regimens to optimize function and limit side effects. From a basic science standpoint, this study could yield valuable insights into the mechanisms of memory encoding and retrieval, taking into account variation in endogenous and exogenous dopamine signaling.

We have previously shown that dopaminergic therapy worsens encoding and improves recall of abstract images in PD (11). This pattern of results is consistent with literature ascribing encoding to VTA-innervated brain regions such as hippocampus and VS, and recall to brain regions including DS (6, 7, 33), which are differentially dopamine depleted in PD (1). Here, we implemented this encoding and retrieval methodology to investigate whether *SLC6A3* gene polymorphisms impact cognition and responses to dopaminergic therapy in PD, and particularly whether these effects are dissimilar for functions mediated by VTA-innervated brain regions vs. DS.

Overall, for PD patients off medication, we expected to see 9R participants outperform 10R/10R participants. On medication, we further predicted that 9R PD patients would be more sensitive to overdose of VTA-innervated brain regions from exogenous dopamine. Hence, 9R carriers were expected to have greatest impairment in memory encoding on relative to off dopaminergic therapy. For recall, mediated by DS and its cortical partners, we expected that all PD patients would recall more items in the ON than OFF dopaminergic state.

## Methods

### Participants

Forty-five patients with PD participated in this study. Patients were diagnosed by a licensed neurologist and met the core assessment criteria for diagnosis of idiopathic PD for surgical interventional therapy and the UK Brain Bank criteria for PD. All patients who participated in this study were referred directly from licensed neurologists. Participants were excluded if they were previously diagnosed with dementia or mild cognitive impairment, if they reported loss of a previous level of function related to cognitive problems, or if they scored less than 22/30 on the Montreal Cognitive Assessment (MOCA). Further, participants were excluded if they were abusing alcohol, prescription or street drugs, or taking medications such as donepezil, galantamine, rivastigmine, memantine, or methylphenidate. Participants were also excluded if they were known to have greater than mild-moderate depression (Beck Depression Inventory score above 30/63) or greater than mild-moderate anxiety (Beck Anxiety Inventory score above 30/63). They were also excluded if they had any other neurological illness. This study was carried out in accordance with the recommendations, and was approved by the ethics review board of both Health Sciences North (Sudbury, Ontario, Canada), and the University of Western Ontario (London, Ontario, Canada). All participants provided written, informed consent in accordance with the Declaration of Helsinki.

Presence as well as severity of disease were assessed for all patients both on and off dopaminergic medication using the motor subscale of the Unified Parkinson's Disease Rating Scale (UPDRS) by a licensed movement disorders neurologist (PAM). All patients were treated with dopamine replacement medications such as dopamine precursors, L-3,4-dihydroxyphenylalanine (L-DOPA), and/or dopamine agonists. Table 1 presents mean group demographic information, screening affective and cognitive measures, and daily doses of DA-replacement medications in L-DOPA equivalents (LED). Calculation of daily LED for each patient was based on theoretical equivalence to L-DOPA (34) as follows: L-DOPA dose + L-DOPA dose x 1/3 if on entacapone + bromocriptine (mg) × 10 + cabergoline or pramipexole (mg) × 67 + ropinirole (mg) × 20 + pergolide (mg) × 100 + apomorphine (mg) × 8.

**Table 1:** Demographic and screening data for PD patients and controls separated by genotype.

	**10R/10R**	**9R**	***p*-value**
*N*	30	14	
Age	68.07 (1.35)	69.14 (1.80)	*ns*
Education	15.13 (0.50)	14.35 (0.86)	*ns*
Years disease	6.83 (1.29)	6.27 (1.30)	*ns*
LED (mg)	683.60 (62.62)	687.63 (86.87)	*ns*
L-DOPA (*n*)	29	14	
DA (*n*)	13	5	
UPDRS (ON)	16.83 (1.00)	16.82 (0.86)	*ns*
UPDRS (OFF)	24.33 (2.10)	22.18 (2.47)	*ns*
COMT Val/Val (*n*)	8	3	
COMT Val/Met (*n*)	16	10	
COMT Met/Met (*n*)	6	1	
BDI-II (ON)	8.00 (1.12)	10.43 (1.34)	*ns*
BDI-II (OFF)	8.43 (0.99)	10.93 (1.51)	*ns*
BAI (ON)	8.60 (1.23)	12.00 (2.63)	*ns*
BAI (OFF)	10.00 (1.48)	11.50 (1.92)	*ns*
SAS (ON)	12.00 (0.98)	12.50 (1.42)	*ns*
SAS (OFF)	11.37 (1.11)	11.50 (1.32)	*ns*
ANART IQ	122.25 (1.50)	123.917 (2.40)	*ns*
F-Words	13.40 (0.74)	16.62 (1.83)	*ns*
A-Words	10.00 (0.79)	13.46 (1.66)	0.04
S-Words	13.30 (0.87)	17.92 (1.99)	0.02
Animals	19.03 (1.08)	19.69 (1.74)	*ns*
MOCA	25.87 (0.42)	26.86 (0.61)	*ns*

All values reported are group means (SEM). Education refers to the number of years spent in the education system. Elaboration of measures used in table follow below.

*Education, years of education; Years disease, years since diagnosis of PD; L-DOPA, L-3,4-dihydroxyphenylalanine; LED, daily L-DOPA equivalent dose in mg; L-DOPA, number of PD patients who were taking L-DOPA, DA, number of PD patients who were taking dopamine agonist drugs; UPDRS, Unified Parkinson's Disease Rating Scale; BDI-II, Beck Depression Inventory II; BAI, Beck Anxiety Inventory; SAS, Starkstein Apathy Scale; ANART IQ, National Adult Reading Test IQ estimation (tested in the ON session only); F-, A-, or S-Words, number of words beginning with the letter F, A, or S, respectively, generated in 60 s (tested in the ON session only); MOCA, total score on the Montreal Cognitive Assessment. Verbal fluency and MOCA tests were completed while on medication*.

### Genotyping procedure

Saliva samples were collected from participants using Oragene 2 mL DNA collection kits (DNA Genotek, Ottawa, Ontario, Canada), and genomic DNA extracted using the MagNA Pure Compact instrument (Roche Diagnostics, Laval, Quebec, Canada). Genotyping of a 40-base pair (bp) variable nucleotide tandem repeat (VNTR; rs28363170) located in the 3'-untranslated region of the *SLC6A3* gene was carried out according to a previously described method (35, 36) with modifications. In brief, a polymerase chain reaction (PCR) was performed (Forward primer: 5'-TGT GGT GTA GGG AAC GGC CTG AG-3', reverse primer: 5'-CTT CCT GGA GGT CAC GGC TCA AGG-3') with the following conditions: initial cycle at 94°C for 5 min, 35 cycles at 94°C for 30 s, 66°C for 30 s, and 72°C for 1 min, and final cycle at 72°C for 7 min. PCR products were loaded on a 6% TBE gel (Invitrogen) to separate a 440-bp and a 480-bp amplicon representing the 9R and 10R alleles, respectively.

Genotyping for the COMT c.472G>A (Val158Met; rs4680) polymorphism was performed using a TaqMan allelic discrimination assay (C__25746809_50; Applied Biosystems®, Foster City, CA, EUA) using 50 ng of genomic DNA per reaction.

One participant was excluded due to an inability to properly determine his/her genotype for the *SLC6A3* gene. As such, 44 PD patients were included in our subsequent analyses.

### Design and procedure

**Figure 1:**
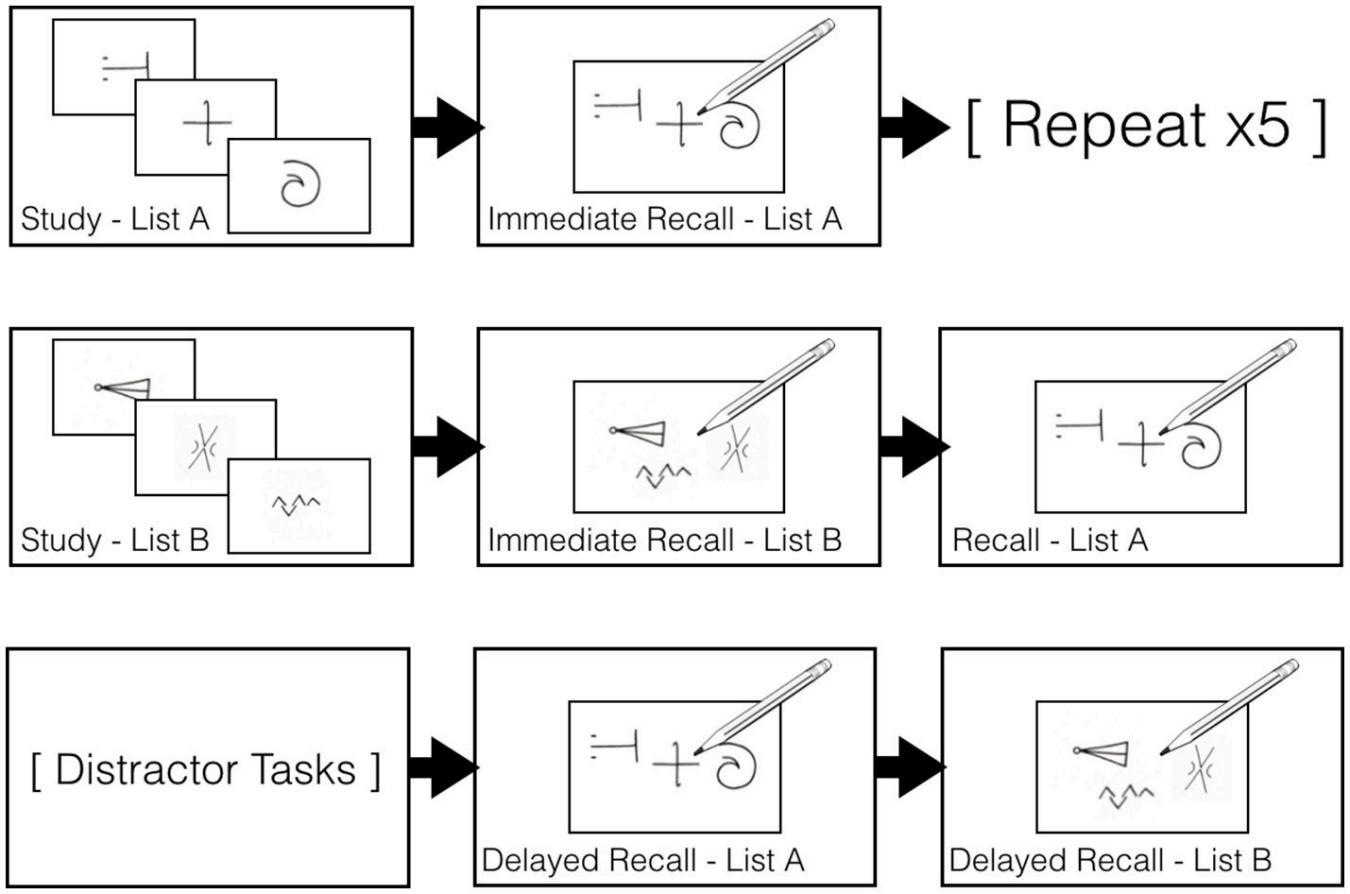
Aggie Figures Learning Test design. *First Row*: The 15 items comprising List A were displayed, one-at-a-time, for 1,000 ms each on a computer screen. After all items had been displayed, participants were given 120 s to draw as many List A items as they could recall. This procedure was repeated five times in total. *Second Row*: The procedure above was completed a single time using the items from List B. Afterward, participants were then given 120 s to draw as many of the List A items they could recall. Third *Row*: Distractor tasks were completed for 30 mins. Participants were then asked to draw as many List A items they could recall. They were then asked to draw as many List B items as they could recall.

All participants performed two versions of the Aggie Figures Learning Test (AFLT) on two consecutive days (11, 37) (Figure 1). PD patients completed the AFLT once while on their usual dopamine-replacement therapy as prescribed by their treating neurologist (i.e., the ON state) and once while they were withdrawn from their dopamine-replacement therapy (i.e., the OFF state). We counterbalanced the ON-OFF order such that half the participants first completed the task while ON and the other half first completed the task while OFF their dopaminergic therapy. In the OFF Session, patients were instructed to abstain from taking L-DOPA for a minimum of 12 to a maximum of 18 h, and dopamine agonists for a minimum of 16 to a maximum of 20 h before testing (Figure 1).

In each session, a set of 15 abstract symbols, List A, was presented to participants. These symbols were presented one at a time for 1,000 ms in the center of a computer monitor. Participants were instructed to try to remember as many of these symbols as possible. After the entire list had been presented, the participant was given 120 s to draw all the symbols that they could remember onto a piece of paper. This study-immediate recall procedure for List A was repeated five times in each version of the AFLT task (Figure 1).

A second set of 15 abstract symbols, List B, was then presented using parameters identical to those above, but List B was only presented a single time. Participants were then given 120 s to draw all the symbols they could remember after the presentation List B. Next, participants were asked to draw all the symbols that they could recall from List A again (Figure 1).

After a 30-min period of delay, during which participants performed distractor tasks (i.e., a number comparison task not reported here), participants were asked to draw all the symbols that they could freely recall from Lists A and B (Figure 1).

### Data analysis

The AFLT was scored by two researchers who were blinded to the identity of the participants (i.e., 10R/10R or 9R) and session (i.e., ON or OFF state). A single point was awarded for each recalled item that could be unambiguously identified. Therefore, items were classified as correct if they had minor distortions in their shape or orientation. Any discrepancies in scoring between the two scorers were addressed such that an agreement was reached concerning scoring of these items.

### Measure of encoding

The difference in the number of correctly recalled items from the first and final study-immediate recall phases was used as our metric of memory encoding (38). That is, the number of items successfully recalled in the first study-immediate recall phase was subtracted from the number of items successfully recalled in the final study-immediate recall phase. This was to control for the effects of working memory and recall abilities. This subtraction serves to better isolate the memory encoding performance. This strategy aims to eliminate effects related to working memory and retrieval abilities on performance, as working memory and retrieval demands are expected to contribute to performance equally for the first and the last study-immediate recall phases, with differences across phases owing more to a participants' ability to encode abstract images into long-term memory (38).

### Measure of retrieval

We used the total number of items recalled from List A after the 30-min delay divided by the total score achieved in the final study-immediate recall phase as our measure of memory retrieval, referred to as Weighted Recall. Unlike study-immediate recall phases, recall after delay is believed to preferentially index retrieval processes (39). Further, by correcting for the number of items recalled on the final study-immediate recall phase, retrieval can be assessed in a less biased manner, controlling for differences between individuals in encoding ability.

### Analyses

Encoding scores and Weighted Recall scores were used as dependent measures in separate 2 × 2 mixed-design analyses of variance (ANOVAs) with Genotype (*SLC6A3* 10R/10R vs. 9R-carriers) as the between-subject variable, and Session (ON vs. OFF) as the within-subject variable. Where warranted by significant interaction results, we followed up with subsequent one-way ANOVAs with Session (ON vs. OFF) as the within-subject factor to explore the simple effects of Session within Genotype.

## Results

### Demographic and clinical data

When examining the effect of *SLC6A3* genotype on our demographic, clinical, and screening measures, we only found significant differences on two of our four measures of verbal fluency. Here, we found that 9R PD patients produced more words beginning with the letters A and S in 60 s than their 10R/10R counterparts. Of note, our tests of verbal fluency were completed while patients were on medication. All other demographic, clinical, and screening measures did not differ between groups (Table 1). It was important to note that our groups did not differ in baseline UPDRS (our measure of disease severity), UPDRS in the ON state (our measure of therapeutic response), disease duration, or LED (our dopaminergic medication dosage). Non-significant differences with respect to disease duration and LED were confirmed with Bayesian analysis in which the Bayes Factors strongly supported the null hypothesis in each case (Disease duration: BF_10_ = 0.329; LED: BF_10_ = 0.337), suggesting that failure to find differences was not the result of lack of power.

### Genotyping results

Thirty participants were homozygous for the 10R allele and 14 participants were carriers of a single 9R allele and a 10R allele. No participants were homozygous for the 9R allele. The *SLC6A3* gene distribution did not deviate from Hardy-Weinberg equilibrium (χ^2^-test, *p* = 1.00).

Additionally, due to the well-documented effects of the *COMT* rs4680 polymorphism on frontal dopamine (40), we also accounted for its distribution in our participants. There was no deviation from Hardy Weinberg equilibrium in the *COMT* rs4680 gene distribution (χ^2^ = 1.62; *p* = 0.20).

### Measure of encoding

**Figure 2:**
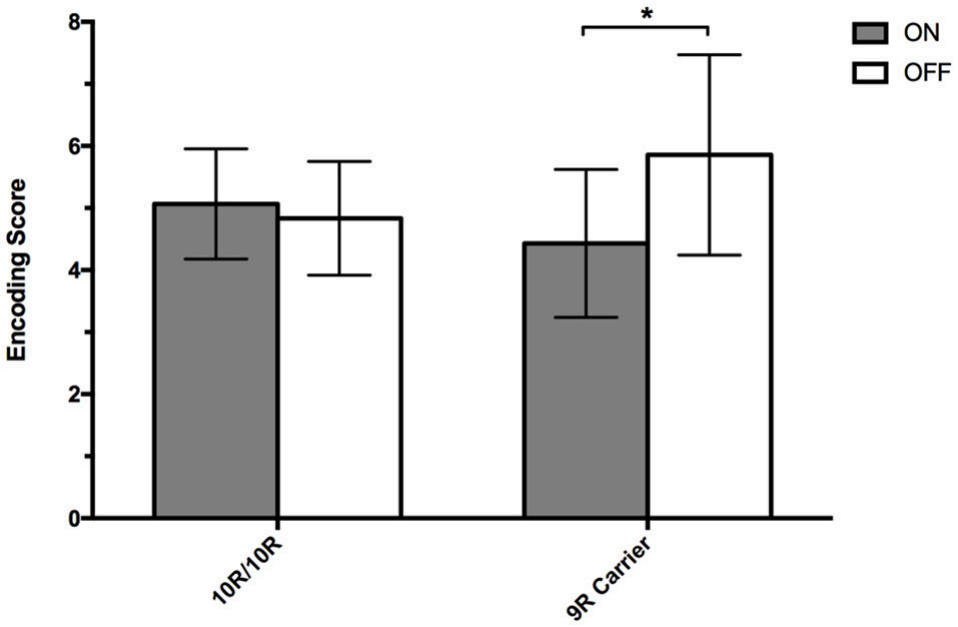
Encoding Scores. Mean encoding scores (± SEM) during the AFLT for PD patients, on and off dopaminergic medication, separated into 9R-carriers (*n* = 14) and 10R/10R-homozygotes (*n* = 30) of the *SLC6A3* 40-bp VNTR polymorphism. Mean encoding scores were calculated by subtracting the number of figures recalled in immediate-recall trial 1 from the number of figures recalled in immediate-recall trial 5. A single asterisk represents *p* = 0.027.

We examined encoding scores in the AFLT in a 2 × 2 mixed ANOVA with *SLC6A3* genotype (*SLC6A3* 10R/10R vs. 9R-carriers) as the between-subject variable, and Session (ON vs. OFF) as the within-subject variable (Table 2; Figure 2). There was no main effect of Session. We found that the Session x *SLC6A3* interaction was significant [*F*_(1, 42)_ = 4.840, *MSE* = 13.182, *p* = 0.033], however. To better understand this interaction, we next examined Session effects for each of the *SLC6A3* genotypes separately. For 9R carrier participants, there was a significant main effect of Session [*F*_(1, 13)_ = 6.250, *MSE* = 14.286, *p* = 0.027]. For 10R/10R participants, there no main effect of Session (*F* < 1). In sum, we see worsening of memory encoding in the ON state (i.e., dopamine overdose effects) in the 9R carrier group only.

**Table 2:** Final study-immediate recall, encoding scores, and weighted recall scores for PD patients separated by *SLC6A3* genotype.

		**Encoding**		**Weighted recall**	
	***n***	**OFF**	**ON**	**OFF**	**ON**
10R/10R	30	4.83 (0.45)	5.07 (0.43)	0.99 (0.05)	1.27 (0.75)
9R	14	5.86 (0.75)	4.43 (0.55)	1.03 (0.09)	1.22 (0.15)

*All values reported are group means (SEM). First trial values correspond to the mean number of items recalled by each group in the first study-immediate recall trial. Final recall values correspond to the mean number of items recalled by each group in the final study-immediate recall trial. Encoding scores were calculated for each participant by subtracting the first recall score from the final recall score. Weighted recall scores were calculated by dividing the number of items recalled after a 30-min delay by the number of items recalled during the final study-immediate recall trial. 10R/10R groups are composed of PD patients who were homozygous for the 10R SLC6A3 40-bp VNTR allele. 9R groups are composed of PD patients who were heterozygous for the 9R SLC6A3 40-bp VNTR allele*.

### Measure of retrieval

**Figure 3:**
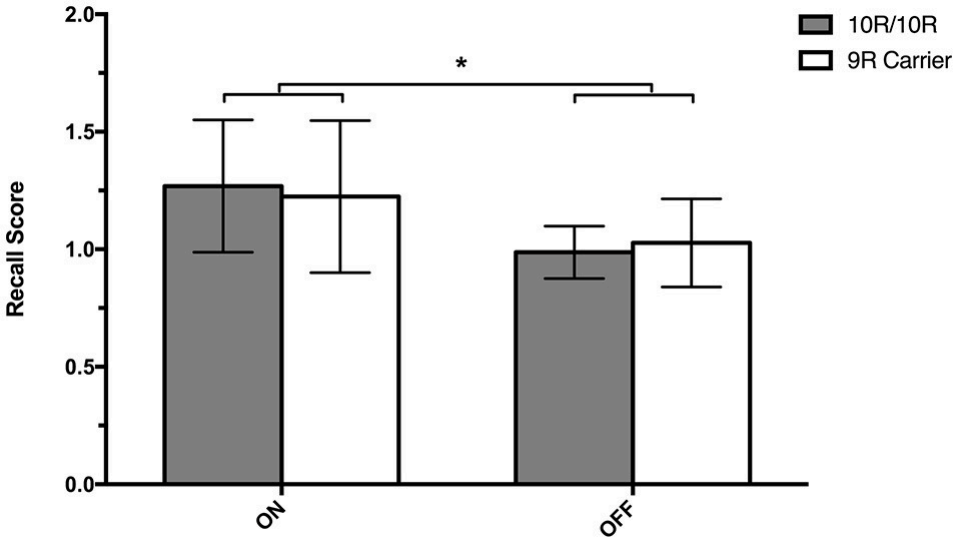
Recall Scores. Mean recall scores (± SEM) during the AFLT for PD patients, on and off medication, separated into 9R-carriers (*n* = 14) and 10R/10R-homozygotes (*n* = 30) of the DAT 40-bp VNTR polymorphism. Mean recall scores were calculated by dividing the number of figures recalled after the 30-min delay by the number of figures recalled during immediate-recall trial 5. A single asterisk represents *p* = 0.040.

We examined Weighted Recall scores in the AFLT in a 2 × 2 mixed ANOVA with *SLC6A3* genotype (*SLC6A3* 10R/10R vs. 9R-carriers) as the between-subject factor and Session (ON vs. OFF) as the within-subject variable (Table 2; Figure 3). We found a significant main effect of Session [*F*_(1, 42)_ = 4.515, *MSE* = 1.099, *p* = 0.040], reflecting better recall performance when on relative to off dopaminergic medication. The Session x *SLC6A3* interaction was not significant (*F* < 1). In summary, we found that the administration of dopamine replacement medication improved recall scores in all PD patients. There were no differential effects related to *SLC6A3* genotype.

## Discussion

### Summary of results

In this study, we investigated whether *SLC6A3* genotype impacted cognition, response to dopaminergic therapy, or both in PD. In PD patients both on and off medication, we differentially assessed encoding of abstract images—a cognitive function mediated by VTA-innervated brain regions (6, 7, 9, 33)—and retrieval of these images—a cognitive function that implicates DS (41)—using the AFLT. We found no main effects of genotype on encoding, but did find a significant interaction between genotype and medication status. In 9R-carriers, dopaminergic therapy worsened encoding scores, relative to performance in the OFF state. No ON-OFF effect was noted in 10R-homozygotes. In this way, the 9R polymorphism appears to predispose to dopamine overdose. We found no main effect of genotype on retrieval, and genotype did not interact with medication status. However, we found that the administration of dopaminergic medication enhanced memory retrieval in PD patients overall.

### Interpretation of memory encoding results

Encoding is consistently shown to recruit (6, 7, 33) and depend upon (42, 43) VTA-innervated brain regions. A large and long-standing literature attributes explicit memory encoding to the VTA-innervated hippocampus (37). VS, another VTA-innervated brain region, is also implicated in memory encoding (6, 7, 11, 41, 42), whether encoding is explicit and intentional (9) or implicit and unintentional (41), even when no reward, punishment, or feedback are present (17, 44, 45).

Compared to the substantially-degenerated SNc, dopamine-producing neurons in the VTA are relatively spared in PD (1). Exogenous dopamine is administered to improve movement abnormalities by remediating dopamine deficiency in the SNc-innervated DS. However, dopaminergic therapy overdoses dopamine-replete VTA-innervated brain regions, such as VS, limbic, and prefrontal cortical regions, impairing their function in PD (2, 3, 12, 13) and in healthy adults (14, 15).

In keeping with these pathophysiological details, whereas PD patients off dopaminergic medication perform comparably to healthy, age-matched controls, PD patients on dopaminergic therapy evidence impaired memory encoding (10, 11, 46, 47). In line with these investigations, we found that dopaminergic therapy worsened memory encoding in PD patients, but only for the carriers of the 9R polymorphism in *SLC6A3* gene. These findings present the intriguing possibility that the 9R *SLC6A3* gene predisposes to dopamine overdose in VTA-innervated brain regions.

### *SLC6A3* effects on encoding on and off dopaminergic therapy in PD

There were no main effects of *SLC6A3* genotype on encoding performance in our study, even though we had predicted superior encoding for PD 9R-carriers relative to 10R homozygotes. As predicted, however, we found that dopaminergic therapy produced greater impairment in encoding for 9R-carriers. In fact, we found no dopaminergic therapy-induced worsening of abstract figure learning in the 10R/10R group. This confirmed our expectations that 9R-carriers are more sensitive to dopamine overdose. Of importance, there were no differences across genotype group in terms of severity of PD (UPDRS OFF), therapeutic response (UPDRS ON-OFF), PD duration, or dopaminergic dosage as expressed by LED to explain differential effect of dopaminergic therapy on encoding.

*SLC6A3* affects re-uptake of dopamine, particularly in striatum and hippocampus. Recent meta-analyses have concluded that expression of *SLC6A3* 9R allele is higher than the 10R-homozygotes (21, 22). As such, 9R-carriers were predicted to have lower dopamine concentrations at baseline due to greater re-uptake. Lower baseline dopamine concentrations arguably yield a higher signal-to-noise ratio, with more impact of event-related, pulsatile dopamine teaching signals. Based on this, we expected superior encoding for 9R compared to 10R homozygotes. We further predicted that this lower baseline dopamine and more optimized signal-to-noise ratio would render 9R-carriers more susceptible to disruption from exogenous dopamine therapy.

Consistent with our finding that PD patients with 9R-carrier status were more susceptible to dopamine overdose of VTA-innervated brain regions, Eisenegger et al. found that following L-DOPA treatment, healthy 9R-carriers were less able than their 10R homozygote counterparts to learn about the playing style of a partner in an interactive, competition-cooperation task (48). As in our study here with PD patients investigating learning of abstract images, in their study in healthy controls, dopaminergic therapy worsened 9R-carriers' ability to learn an adaptive strategy to maximize their winnings (48).

### Interpretation of memory retrieval results

Explicit retrieval processes implicate more distributed brain regions compared to encoding. Some of the brain regions implicated in retrieval overlap with encoding, such as hippocampus (7, 49), but the DS and cortical regions to which DS is reciprocally connected are also involved. In patients with DS lesions, explicit memory is commonly impaired (50, 51). Frontal lobe lesions, particularly in dorsolateral prefrontal cortex, which is an important cortical partner of DS, also commonly impair free recall (52, 53). Further, regions such as the dorsal frontoparietal network are engaged preferentially during free recall (49).

DS is seriously dopamine restricted in PD at baseline, even at early stages of disease. Functions performed by DS and its cortical partners are consistently improved by dopaminergic supplementation (3, 11). In the current study we replicated the finding that PD patients' recall ability is improved with the administration of dopaminergic therapy (11). We also found that for retrieval, unlike encoding, the effect of dopaminergic therapy was not modulated by genotype.

### *SLC6A3* effects on recall on and off dopaminergic therapy in PD

The predictions regarding the effect of SLCA3 gene on recall were less clear than they were for encoding. Although more optimized signal-to-noise ratio in 9R-carriers might be expected to benefit recall performance, features of DS DAT concentration and dopamine regulation, as well as previous findings, made the predicted effects of SCLA3 gene on recall performance more complex. The cytoarchitectonics of DS are substantially different from those of VS. The high density of dopamine inputs on DS MSNs cause rapid, maximal responses through a wide range of firing frequencies and intensities (54, 55). Further, DS contains extremely high concentrations of DAT, resulting in short periods of dopaminergic stimulation (54). Therefore, dopaminergic stimulation in DS seems to produce a binary signal because brief and maximal responding occurs over very wide ranges of stimulation. Based on this, it seemed unlikely that subtle differences in *SLC6A3* expression and small variations in signal-to-noise ratio related to *SLC6A3* gene polymorphisms would significantly impact DS function.

Finally, we also expected that the effect of *SLC6A3* genotype would be relatively negligible in the face of the substantial dopamine deficiency to DS that occurs at all stages of disease in PD. Motor and cognitive functions mediated by DS and its cortical partners are markedly impaired in the off state and are improved with dopaminergic therapy (2–4, 17). Consequently, we expected PD patients would recall more items on relative to off medication irrespective of *SLC6A3* genotype. This prediction was borne out.

## Conclusion

To our knowledge, this is the first investigation of the effect of *SLC6A3* genotype on (a) cognitive functions mediated by VTA-innervated brain regions vs. DS, and (b) response to dopaminergic therapy in PD. Replicating our previous findings (11), dopaminergic therapy differentially affected explicit memory encoding and retrieval in PD patients. Dopaminergic therapy *improved* recall performance in PD patients irrespective of *SLC6A3* genotype, whereas it *impaired* encoding, but only for 9R-carriers. This pattern of findings is in keeping with the view that encoding is mediated by dopamine-replete VTA-innervated brain regions, such as hippocampus and VS, whereas recall is mediated by broad brain regions including the significantly dopamine-deplete DS and its cortical partners. These results indicate that whereas dopaminergic therapy benefits DS function in all PD patients irrespective of *SLC6A3* genotype, 9R-carrier status predisposes to dopamine overdose of VTA-innervated brain regions. We speculate that higher *SLC6A3* expression in 9R allele carriers, and consequently lower basal dopamine, yields a more optimized signal-to-noise dopamine ratio that is more vulnerable to disruption by exogenous dopamine. This is in comparison to 10R homozygotes who are adapted to higher and more variable baseline, tonic dopamine. These results suggest that 9R-carrier status predisposes to dopamine overdose.

## Author contributions

PM, RK, KS, BR, and NH designed the experiment. BR, AA, AM, NH, US, RK, and RT acquired and analyzed data. BR and PM wrote the manuscript. All authors edited the manuscript.

### Conflict of interest statement

The authors declare that the research was conducted in the absence of any commercial or financial relationships that could be construed as a potential conflict of interest.
